# Trends in prevalence and incidence of scabies from 1990 to 2017: findings from the global Burden of disease study 2017

**DOI:** 10.1080/22221751.2020.1754136

**Published:** 2020-04-26

**Authors:** Wei Zhang, Yujiao Zhang, Lina Luo, Wenyong Huang, Xiaoping Shen, Xian Dong, Wen Zeng, Hongguang Lu

**Affiliations:** a Department of Dermatology, Affiliated Hospital of Guizhou Medical University, Guiyang, People’s Republic of China; b School of Public Health, Guizhou Medical University, Guiyang, People’s Republic of China

**Keywords:** Scabies, prevalence, incidence, global burden of disease, trend analysis

## Abstract

Scabies remains a significant public health concern globally, affecting people of all ages, races, and socioeconomic groups. The epidemic characteristics of scabies are yet unravelled, especially in high-income countries and elderly population. In this study, we sought to investigate incidence and prevalence and their trends of scabies worldwide from 1990 to 2017 via the Global Burden of Disease (GBD) Study 2017. The prevalence and incidence of scabies tend to a moderate increase after age 70. The annual percentage change in ASR of scabies prevalence and incidence increased in high SDI countries and High-income North America. Current prevention strategies should be reoriented, and much more targeted strategies should be established in some populations to forestall the increase in scabies.

Scabies is an ancient skin infestation worldwide, and yet it has recently been becoming a renewed global concern due to its unprecedented social cost of this erstwhile disease [1]. The development of comprehensive scabies control strategies has been constrained in the past decade due to the scarcity of global epidemiology data [2]. In previous studies, a global systematic review of prevalence studies of scabies by searching the published studies in a community setting was reported [3], however, the limitation of the available data was the methodological heterogeneity in this study. Another global research mainly seemed to focus on a cross-sectional analysis of the global burden of scabies using the disability-adjusted life-years (DALYs) metric, indicating high prevalence of scabies in hot, tropical areas [4]. Moreover, although this disease mainly occurs in resource-poor tropical settings, particularly among children, it also outbreaks in population of elderly people in high-income regions [5]. The global epidemic characteristics of scabies remain to be unravelled, especially in high-income countries and elderly population. We, therefore, aimed to investigate the pattern and temporal trends of scabies incidence and prevalence worldwide from 1990 to 2017 for guiding scabies control measures.

Based on the Global Burden of Disease study (GBD) 2017 [4], we collected detailed data on scabies epidemiology (case number and age-standardized rate [ASR]) between 1990 and 2017 according to sex, Socio-demographic Index (SDI), 195 countries and territories, and twenty age categories (5-year groups within ages 0–94 years, and ≥95 years). These countries and territories were separated into 5 regions according to SDI, including low, low-middle, middle, high-middle, and high SDI. In terms of the geography of the world, it was categorized into 21 regions, e.g. High-income North America (Supplementary Table 1). The Detailed methods of estimations of scabies incidence and prevalence have been previously reported [4]. Brieﬂy, the scabies cases were defined by the International Classification of Diseases (ICD)-9 code 133 and ICD-10 code B86 [4]. The Bayesian meta-regression disease modelling tool, DisMod-MR 2.1 [4], was used to estimate all data above and its 95% uncertainty intervals (UIs) by all locations, both sexes, all age groups. Furthermore, annual percentage changes (APCs) in scabies ASR were calculated to quantify the temporal trends in prevalence and incidence of scabies worldwide. A regression line model was applied to describe the APCs in ASR, fitting the natural logarithm of the rates i.e. *y* = *α* *+* *βx* *+* *ϵ*, where *y* = ln(ASR), and *x* = calendar year. The APCs in ASR were estimated as 100 × (exp(*β*)−1), and captured 95% conﬁdence interval (CI) [4,6]. All statistics were analysed via R 3.5.2. A *p* value of less than 0.05 was considered statistically signiﬁcant.

In 2017, the global prevalence and incidence cases of scabies in both sexes were 175.4 million (95%UI 154.5–198.4) and 527.5 million (95%UI 462.1–598.1), respectively (Supplementary Table 1). The prevalence and incidence pattern were heterogeneous across 195 regions and countries (Supplementary Table 1; Figure 1(A,B)). The most pronounced number of prevalence and incidence were generally observed in tropical regions and countries with a low SDI, such as Papua New Guinea (ASR = 5620.2 and 16,763.7 per 100,000 population, respectively). Moreover, scabies prevalence and incidence were substantially higher in children than in adolescents and adults. The age-specific rate of scabies increased sharply from age 5 to age 25, and was on the decline after age 25, but with a moderate increase again after age 70 (Figure 2). Globally, the ASR of prevalence and incidence of scabies in both sexes decreased by 7.40% and 7.47% from 1990 to 2017 (Supplementary Figure 1), with the APC of −0.26 (95% CI −0.27 to −0.25) and −0.27 (95% CI −0.28 to −0.26), respectively (Supplementary Table 1). However, from 1990 to 2017, for all ages and both sexes combined, the APC of prevalence and incidence in ASR increased in high SDI countries (0.51; 95%CI 0.49–0.54) and High-income North America (0.48; 95%CI 0.36–0.59), such as United States (0.51; 95%CI 0.38–0.65) and (0.53; 95%CI 0.4–0.67), which were more remarkable than that in other countries in the same period (Supplementary Table 1; Figure 1(C,D)).

**Figure 1.**
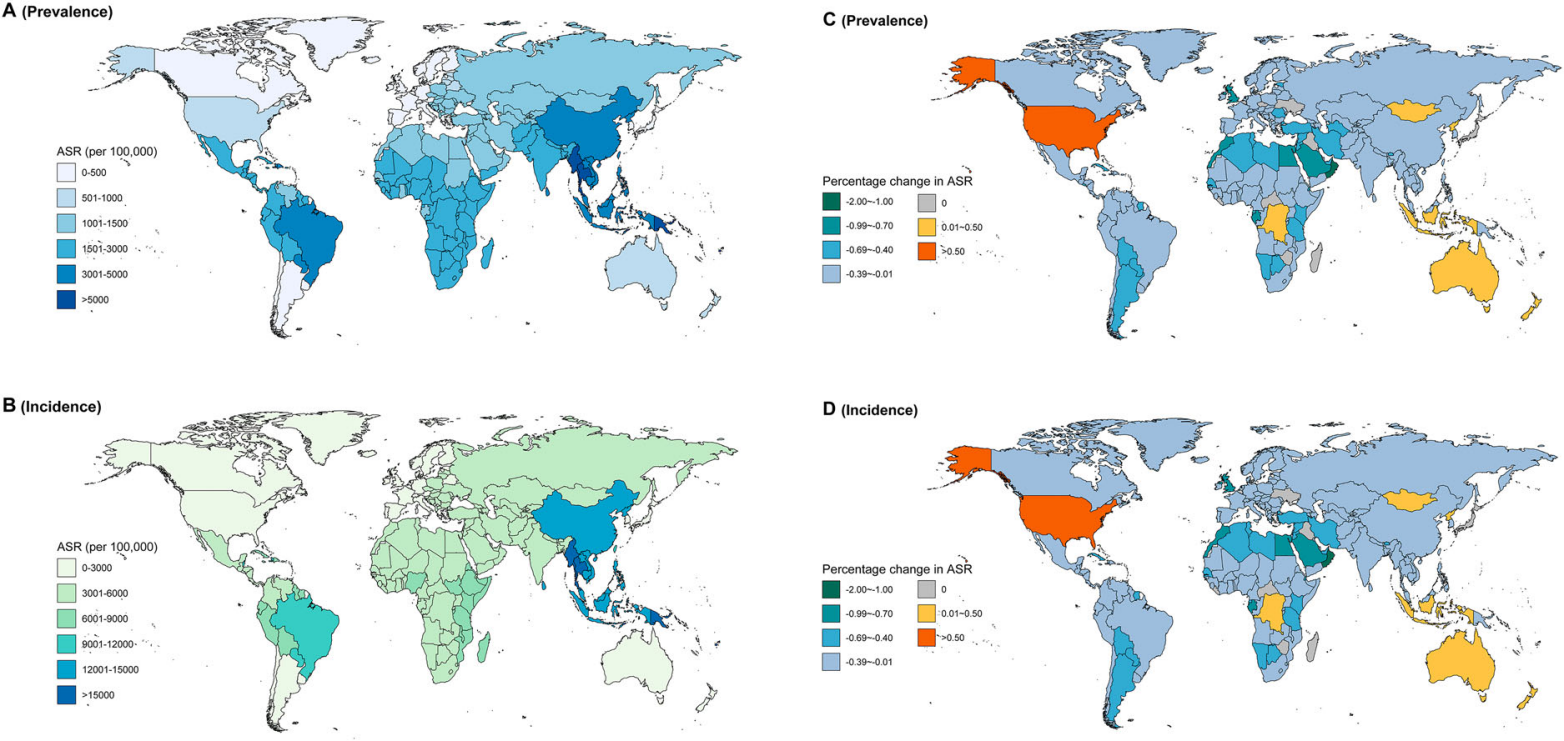
The global distribution of age standardized rate (ASR) and the corresponding annual percentage change (APC) of scabies prevalence and incidence for both sexes. (A) The ASR of scabies prevalence in 2017; (B) The ASR of scabies incidence in 2017; (C) The APC in ASR of scabies prevalence from 1990 to 2017; (D) The APC in ASR of scabies incidence from 1990 to 2017.

**Figure 2.**
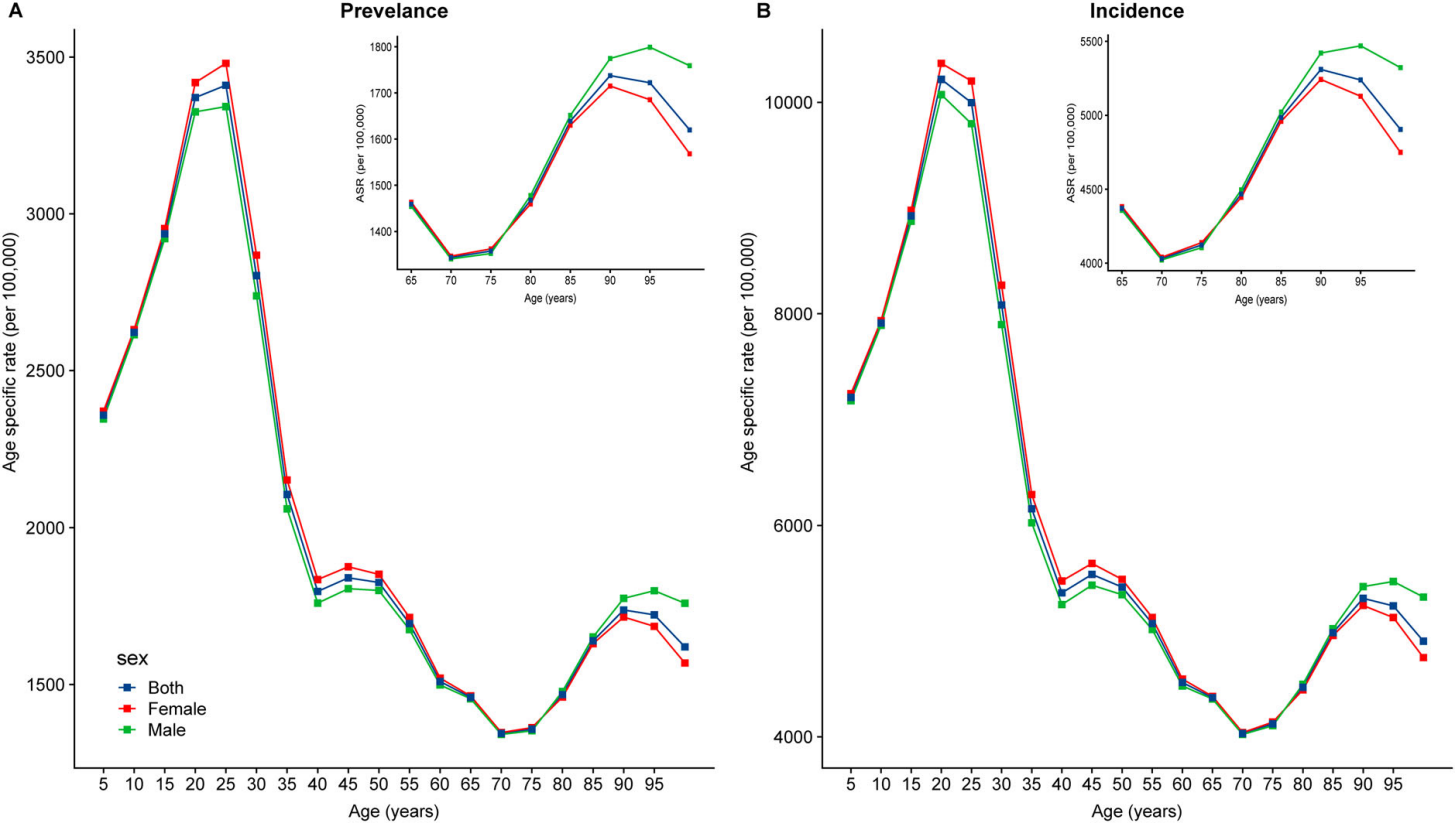
The global age specific rate of scabies prevalence (A) and incidence (B) for both sexes in 2017.

Our study has shown that overall, scabies prevalence and incidence were on a downwards trend in the worldwide from 1990 to 2017(Supplementary Figure 1). The effective strategy of mass drug administration for scabies in resource-limited island settings may be one of the important contributions [7], which attributed to the descent tendency of global epidemic situation of scabies and decreased prevalence of scabies. The prevalence and incidence were substantially high in children, in the low SDI and tropical regions in accordance with previous studies [1,3,4], suggesting that environmental or socio-economic and political factors such as population crowding, inadequate medical therapy, and hot and humid climates increase the scabies epidemics [2]. Although the prevalence and incidence of scabies increased sharply from age 5 to age 25, and was on the decline after age 25, its epidemics in elderly population (70–90 years of age) presented a rising trend again. Moreover, we observed an unexpected trend of the APC of prevalence and incidence in ASR in such countries with high SDI as the United States and Australia where scabies prevalence and incidence were higher than those in other high SDI countries, even other many resource-poor tropical countries from 1990 to 2017. The estimated APC in ASR, a summary and widely used measure of the ASR trend over a speciﬁed interval [6], which can quantify the scabies prevalence and incidence trends. This is likely related to increasing population density in urban settings, migrants and ageing population [1]. For instance, scabies outbreaks in residential and nursing care homes for elderly people are becoming common with growing numbers of the elderly worldwide [5]. As a severe form, crusted scabies mainly occurs in the elderly people or those with weakened immune systems, which is difficult to diagnose and with greater complications [8–10]. Therefore, the prevalence and incidence of scabies might be underreported in GBD, and more attention should be paid to scabies control among these groups.

Scabies transmission occurs with direct person-to-person contact or through items that carry the mites, such as bedding and clothing. Thus, it is important to wash all bedding, clothes, and towels in hot water and dry them by the hot cycle. If some objects could not be washed, these can be decontaminated via isolating them from human skin in a sealed plastic bag for 1 week [8,9]. Additionally, all people living in the same household with the patient need to be treated [10].

Some limitations of the GBD 2017 estimates on scabies should be noted. Although the GBD fills a gap in which actual data on disease epidemiology are sparse or unavailable, underreporting of scabies is an objective fact. For example, the underreporting of scabies due to the immigration phenomena is one of the main issues in GBD scabies data. The GBD analysis data on the special immunosuppressed population due to scabies are not covered. Additionally, the GBD analysis of relevant scabies data on clinical subtypes, risk factors, and complications such as impetigo, glomerulonephritis and rheumatic fever are also limited. Therefore, the temporal trends in prevalence and incidence of scabies stratified by these factors were not estimated in our study.

In conclusion, despite the temporal trends of prevalence and incidence due to scabies globally declined over the past 27 years, there were undesirable increases in the high SDI, High-income North America regions and in elderly population. Current prevention strategies should be reoriented, and much more targeted and specific strategies should be established in some countries and regions to forestall the increase in scabies such as the elderly population.

## Supplementary Material

Supplemental Material

## Data Availability

The data used to support the findings of this study were extracted from the GBD 2017 database, which is free available on http://ghdx.healthdata.org/gbd-results-tool.
