# Supplementary material for: Trends in prevalence and incidence of scabies from 1990 to 2017: findings from the global Burden of disease study 2017
Source: Emerg Microbes Infect. 2020 Apr 26;9(1):813–6. doi: 10.1080/22221751.2020.1754136 (PMC7241492; doi:10.1080/22221751.2020.1754136)
Supplement: Supplemental Material [file TEMI_A_1754136_SM0940.docx]

**Supplemental Materials：**

**Title: Trends in Prevalence and Incidence of Scabies from 1990 to 2017: Findings from the Global Burden of Disease Study 2017**

Wei Zhang^1^ M.D., Yujiao Zhang^1^ M.D., Lina Luo^1^ M.D., Wenyong Huang^2^ Ph.D., Xiaoping Shen^1^ M.D., Xian Dong^1^ M.D., Wen Zeng^1^ M.D., Hongguang Lu^1^* M.D., Ph.D.

^1^ Department of Dermatology, Affiliated Hospital of Guizhou Medical University, Guiyang, Guizhou 550001, P.R. China

^2^ School of Public Health, Guizhou Medical University, Guiyang, Guizhou 550001, P.R. China

*Corresponding author:

Hongguang Lu

Postal address: Department of Dermatology, Affiliated Hospital of Guizhou Medical University. No.28 Guiyi Road, Guiyang, Guizhou, 550001, P.R. China;

E-mail: [cnsnlj@outlook.com](mailto:cnsnlj@outlook.com); hongguanglu@hotmail.com

Tel: +8613984120866; +86-851-86752685; Fax: +86-851-86820346

**Running head:** Prevalence and Incidence of Scabies Worldwide.

**Keywords:** Scabies; prevalence; incidence; global burden of disease; trend analysis

Table S1, Supplementary Table 1; Figure S1, Supplementary Figure 1.

**Table S1. Scabies prevalence and incidence in 2017 and the temporal trends from 1990 to 2017**

|  | **Characteristics** | **Prevalence** | | |  | **Incidence** | | |  |
| --- | --- | --- | --- | --- | --- | --- | --- | --- | --- |
|  |  | Numbers  (*1000) (95% UI) | ASR  No. (95% UI) | Percentage change in ASR  (95% CI) |  | Numbers  (*1000) (95% UI) | ASR  No. (95% UI) | Percentage  change in ASR  (95% CI) |  |
|  | **Global** |  |  |  |  |  |  |  |  |
|  | Both | 175406.7 | 2322.4 | -0.26 |  | 527476.5 | 6995.0 | -0.27 |  |
|  |  | (154517.9-198404.1) | (2045.9-2630.3) | (-0.27- -0.25) |  | (462050.9-598087.9) | (6133.2-7942.1) | (-0.28- -0.26) |  |
|  | Male | 87419.4 | 2294.4 | -0.29 |  | 262992.7 | 6912.0 | -0.29 |  |
|  |  | (77029.2-99080.9) | (2020.9-2593.3) | (-0.30- -0.28) |  | (229939.1-298371.4) | (6053.3-7848.5) | (-0.30- -0.28) |  |
|  | Female | 87987.3 | 2351.5 | -0.24 |  | 264483.8 | 7081.1 | -0.25 |  |
|  |  | (77356.5-99253.1) | (2066.0-2668.3) | (-0.25- -0.23) |  | (231847.6-299573.0) | (6194.5-8037.3) | (-0.26- -0.24) |  |
|  | **High SDI** |  |  |  |  |  |  |  |  |
|  | Both | 6202.9 | 610.8 | 0.53 |  | 18615.2 | 1840.9 | 0.51 |  |
|  |  | (5595.0-6890.8) | (544.0-682.6) | (0.50-0.56) |  | (16730.4-20583.0) | (1633.3-2068.4) | (0.49-0.54) |  |
|  | Male | 3031.1 | 603.8 | 0.52 |  | 9098.1 | 1820.3 | 0.50 |  |
|  |  | (2725.5-3371.5) | (537.4-675.9) | (0.49-0.54) |  | (8167.6-10099.8) | (1616.4-2044.1) | (0.48-0.53) |  |
|  | Female | 3171.8 | 617.8 | 0.53 |  | 9517.2 | 1861.5 | 0.52 |  |
|  |  | (2860.4-3519.2) | (550.3-688.6) | (0.50-0.56) |  | (8562.7-10527.2) | (1651.6-2084.9) | (0.49-0.55) |  |
|  | **High-middle SDI** |  |  |  |  |  |  |  |  |
|  | Both | 34799.4 | 2630.5 | -0.22 |  | 104721.6 | 7960.3 | -0.22 |  |
|  |  | (30780.4-39251.0) | (2316.4-2987.4) | (-0.25- -0.19) |  | (92738.3-117513.8) | (6973.8-9030.3) | (-0.25- -0.20) |  |
|  | Male | 17705.2 | 2639.3 | 3.10 |  | 53285.4 | 7987.7 | 3.10 |  |
|  |  | (15579.4-19994.9) | (2320.6-2998.3) | (0.54-5.73) |  | (47113.5-59797.2) | (7008.3-9060.9) | (0.54-5.73) |  |
|  | Female | 17094.2 | 2623.9 | -0.20 |  | 51436.3 | 7940.0 | -0.20 |  |
|  |  | (15155.5-19276.3) | (2307.5-2975.4) | (-0.23- -0.17) |  | (45649.6-57769.6) | (6959.9-9012.8) | (-0.23- -0.17) |  |
|  | **Middle SDI** |  |  |  |  |  |  |  |  |
|  | Both | 65715.9 | 3206.1 | -0.48 |  | 197983.8 | 9691.9 | -0.47 |  |
|  |  | (57870.4-73912.2) | (2827.9-3632.9) | (-0.49- -0.46) |  | (173952.9-224564.7) | (8518.4-10963.8) | (-0.49- -0.46) |  |
|  | Male | 32915.9 | 3181.0 | -0.49 |  | 99203.9 | 9616.2 | -0.48 |  |
|  |  | (29027.1-37030.9) | (2806.0-3598.9) | (-0.50- -0.47) |  | (87034.6-112644.2) | (8446.2-10895.6) | (-0.49- -0.46) |  |
|  | Female | 32800.0 | 3229.5 | -0.47 |  | 98779.9 | 9762.7 | -0.47 |  |
|  |  | (28975.9-36857.4) | (2846.8-3660.3) | (-0.49- -0.46) |  | (86945.1-111596.9) | (8569.6-11035.1) | (-0.48- -0.45) |  |
|  | **Low-middle SDI** |  |  |  |  |  |  |  |  |
|  | Both | 39651.7 | 2236.4 | -0.36 |  | 118962.4 | 6706.8 | -0.37 |  |
|  |  | (34480.8-45374.8) | (1967.9-2528.9) | (-0.37- -0.35) |  | (103292.5-136942.2) | (5873.7-7634.9) | (-0.38- -0.36) |  |
|  | Male | 19442.9 | 2175.2 | -0.36 |  | 58376.9 | 6526.0 | -0.37 |  |
|  |  | (16893.9-22226.7) | (1913.7-2465.4) | (-0.37- -0.35) |  | (50674.1-67236.5) | (5727.2-7435.1) | (-0.38- -0.35) |  |
|  | Female | 20208.7 | 2297.3 | -0.37 |  | 60585.5 | 6887.0 | -0.37 |  |
|  |  | (17587.4-23153.3) | (2025.5-2602.9) | (-0.38- -0.36) |  | (52630.6-69708.3) | (6039.8-7843.7) | (-0.38- -0.36) |  |
|  | **Low SDI** |  |  |  |  |  |  |  |  |
|  | Both | 27981.7 | 1996.0 | -0.14 |  | 84019.8 | 5971.5 | -0.14 |  |
|  |  | (32660.6-23999.0) | (1740.1-2269.5) | (-0.16- -0.12) |  | (72313.4-97923.5) | (5204.1-6832.6) | (-0.16- -0.12) |  |
|  | Male | 13789.1 | 1948.5 | -0.12 |  | 41418.2 | 5828.6 | -0.13 |  |
|  |  | (11804.8-16078.4) | (1700.8-2214.5) | (-0.12- -0.15) |  | (35553.9-48151.7) | (5075.1-6652.9) | (-0.16- -0.11) |  |
|  | Female | 14192.6 | 2044.3 | -0.15 |  | 42601.6 | 6116.7 | -0.16 |  |
|  |  | (12188.8-16539.1) | (1780.0-2330.0) | (-0.17- -0.14) |  | (36691.5-49830.8) | (5326.4-7021.9) | (-0.17- -0.14) |  |
|  | **Regional** |  |  |  |  |  |  |  |  |
|  | High-income Asia Pacific | |  |  |  |  |  |  |  |
|  | Both | 438.1 | 283.1 | -0.02 |  | 1315.4 | 854.2 | -0.01 |  |
|  |  | (386.4-492.1) | (245.5-325.6) | (-0.03-0.00) |  | (1164.4-1476.4) | (742.2-983.9) | (-0.03-0.00) |  |
|  | Male | 210.1 | 280.3 | -0.10 |  | 630.2 | 845.7 | -0.10 |  |
|  |  | (184.7-237.5) | (241.9-323.1) | (-0.12- -0.08) |  | (557.0-707.8) | (729.5-979.1) | (-0.12- -0.08) |  |
|  | Female | 228.1 | 285.7 | 0.07 |  | 685.2 | 862.4 | 0.07 |  |
|  |  | (202.1-255.9) | (247.1-326.7) | (0.05-0.10) |  | (609.1-766.6) | (751.9-994.2) | (0.05-0.10) |  |
|  | East Asia |  |  |  |  |  |  |  |  |
|  | Both | 56686.2 | 4054.0 | -0.32 |  | 170580.4 | 12271.5 | -0.32 |  |
|  |  | (50208.8-63899.4) | (3569.5-4596.4) | (-0.34- -0.31) |  | (151074.9-191155.7) | (10782.3-13890.2) | (-0.33--0.30) |  |
|  | Male | 28834.9 | 4043.4 | -0.32 |  | 86812.1 | 12239.0 | -0.32 |  |
|  |  | (25465.2-32533.2) | (3559.0-4584.9) | (-0.34- -0.31) |  | (76947.6-97233.5) | (10762.6-13853.0) | (-0.34- -0.31) |  |
|  | Female | 27851.3 | 4060.6 | -0.32 |  | 83768.3 | 12292.1 | -0.32 |  |
|  |  | (24659.1-31363.9) | (3572.9-4594.5) | (-0.33--0.31) |  | (74251.5-93946.2) | (10799.3-13912.9) | (-0.33--0.30) |  |
|  | Southeast Asia |  |  |  |  |  |  |  |  |
|  | Both | 31692.6 | 4803.5 | -0.09 |  | 95508.6 | 14513.0 | -0.08 |  |
|  |  | (27978.3-35823.0) | (4248.2-5428.2) | (-0.11- -0.08) |  | (84057.9-108413.5) | (12772.6-16421.0) | (-0.10- -0.07) |  |
|  | Male | 15778.0 | 4767.1 | -0.09 |  | 47554.0 | 14402.4 | -0.09 |  |
|  |  | (13906.1-17852.8) | (4211.3-5405.2) | (-0.11- -0.08) |  | (41826.2-54032.3) | (12673.6-16329.1) | (-0.10- -0.07) |  |
|  | Female | 15914.6 | 4837.4 | -0.09 |  | 47954.5 | 14615.9 | -0.08 |  |
|  |  | (14034.5-17971.3) | (4273.9-5468.2) | (-0.10- -0.07) |  | (42345.4-54315.8) | (12852.3-16551.2) | (-0.10- -0.06) |  |
|  | South Asia |  |  |  |  |  |  |  |  |
|  | Both | 33364.6 | 1801.1 | -0.10 |  | 100168.337 | 5415.9 | -0.11 |  |
|  |  | (28905.2-38586.3) | (1566.2-2057.0) | (-0.15- -0.05) |  | (86470.398 116052.013) | (4708.4-6207.4) | (-0.15- -0.06) |  |
|  | Male | 16700.3 | 1758.9 | -0.10 |  | 50154.5 | 5289.2 | -0.11 |  |
|  |  | (14455.3-19317.2) | (1532.2-2008.8) | (-0.16- -0.05) |  | (43278.5-58110.5) | (4598.2-6062.3) | (-0.16- -0.06) |  |
|  | Female | 16664.3 | 1845.5 | -0.10 |  | 50013.8 | 5549.4 | -0.11 |  |
|  |  | (14430.1-19201.1) | (1604.9-2109.6) | (-0.15- -0.06) |  | (43187.4-57992.1) | (4817.7-6378.2) | (-0.15- -0.06) |  |
|  | Central Asia |  |  |  |  |  |  |  |  |
|  | Both | 1019.8 | 1120.2 | -0.15 |  | 3079.6 | 3388.9 | -0.15 |  |
|  |  | (882.9-1173.2) | (969.9-1290.2) | (-0.17- -0.14) |  | (2673.6-3529.9) | (2936.6-3885.7) | (-0.17- -0.13) |  |
|  | Male | 488.9 | 1071.1 | -0.14 |  | 1477.4 | 3241.4 | -0.14 |  |
|  |  | (423.4-559.7) | (930.2-1226.6) | (-0.15- -0.13) |  | (1281.8-1694.6) | (2824.1-3708.9) | (-0.15- -0.12) |  |
|  | Female | 530.9 | 1169.2 | -0.16 |  | 1602.2 | 3536.2 | -0.16 |  |
|  |  | (460.7-611.5) | (1011.5-1348.6) | (-0.18- -0.14) |  | (1388.2-1840.7) | (3064.3-4077.9) | (-0.18- -0.13) |  |
|  | Oceania |  |  |  |  |  |  |  |  |
|  | Both | 705.7 | 5478.0 | -0.11 |  | 2114.0 | 16367.4 | -0.13 |  |
|  |  | (622.5-798.7) | (4876.4-6127.5) | (-0.14- -0.09) |  | (1856.1-2405.9) | (14573.0-18371.4) | (-0.15- -0.10) |  |
|  | Male | 367.2 | 5519.2 | -0.12 |  | 1100.2 | 16488.5 | -0.13 |  |
|  |  | (322.8-414.8) | (4914.8-6175.0) | (-0.14- -0.09) |  | (964.4-1256.0) | (14671.2-18502.4) | (-0.15- -0.11) |  |
|  | Female | 338.5 | 5428.9 | -0.11 |  | 1013.8 | 16223.4 | -0.13 |  |
|  |  | (295.9-382.7) | (4800.4-6105.4) | (-0.14- -0.09) |  | (889.9-1147.6) | (14421.7-18189.5) | (-0.15- -0.10) |  |
|  | Western Europe |  |  |  |  |  |  |  |  |
|  | Both | 340.3 | 95.1 | -0.30 |  | 1018.5 | 286.2 | -0.30 |  |
|  |  | (299.3-385.5) | (82.1-110.1) | (-0.36- -0.24) |  | (898.9-1148.649) | (248.1-331.7) | (-0.35- -0.24) |  |
|  | Male | 169.5 | 94.3 | -0.25 |  | 507.7 | 283.7 | -0.24 |  |
|  |  | (148.7-191.9) | (81.6-109.2) | (-0.29- -0.21) |  | (447.6-572.0) | (247.0-327.1) | (-0.28- -0.20) |  |
|  | Female | 170.7 | 96.1 | -0.35 |  | 510.9 | 289.2 | -0.35 |  |
|  |  | (150.2-193.7) | (83.0-111.6) | (-0.43- -0.27) |  | (452.7-578.4) | (251.5-336.8) | (-0.44- -0.27) |  |
|  | Central Europe |  |  |  |  |  |  |  |  |
|  | Both | 1022.5 | 1030.0 | -0.16 |  | 3074.7 | 3114.1 | -0.16 |  |
|  |  | (903.7-1152.8) | (892.5-1183.4) | (-0.18- -0.15) |  | (2708.6-3458.6) | (2695.7-3572.9) | (-0.17- -0.14) |  |
|  | Male | 478.0 | 976.2 | -0.16 |  | 1437.3 | 2952.1 | -0.15 |  |
|  |  | (419.1-540.7) | (847.5-1119.8) | (-0.17- -0.14) |  | (1264.3-1628.4) | (2554.4-3394.4) | (-0.17- -0.14) |  |
|  | Female | 544.5 | 1084.9 | -0.16 |  | 1637.4 | 3279.2 | -0.16 |  |
|  |  | (483.2-612.4) | (939.7-1246.0) | (-0.18- -0.14) |  | (1450.1-1833.7) | (2850.5-3760.4) | (-0.18- -0.14) |  |
|  | Eastern Europe | |  |  |  |  |  |  |  |
|  | Both | 2096.7 | 1151.3 | -0.03 |  | 6298.6 | 3476.0 | -0.03 |  |
|  |  | (1847.1-2370.7) | (997.1-1325.9) | (-0.05- -0.02) |  | (5560.6-7111.2) | (3017.3-4016.1) | (-0.05- -0.02) |  |
|  | Male | 1089.3 | 1249.4 | -0.05 |  | 3271.0 | 3771.0 | -0.05 |  |
|  |  | (953.2-1235.9) | (1077.2-1440.4) | (-0.07- -0.04) |  | (2865.2-3707.2) | (3276.0-4354.2) | (-0.07- -0.04) |  |
|  | Female | 1007.4 | 1056.1 | -0.02 |  | 3027.6 | 3189.3 | -0.02 |  |
|  |  | (890.3-1134.5) | (913.6-1218.8) | (-0.04-0.00) |  | (2679.1-3418.4) | (2760.4-3694.7) | (-0.04-0.00) |  |
|  | North Africa and Middle East | |  |  |  |  |  |  |  |
|  | Both | 8024.0 | 1304.4 | -0.48 |  | 24030.0 | 3909.5 | -0.47 |  |
|  |  | (6971.8-9162.1) | (1137.2-1484.9) | (-0.50- -0.45) |  | (20834.6-27481.3) | (3400.5-4458.4) | (-0.50- -0.45) |  |
|  | Male | 4052.7 | 1272.4 | -0.48 |  | 12138.9 | 3814.8 | -0.47 |  |
|  |  | (3535.8-4648.0) | (1114.0-1451.8) | (-0.51- -0.45) |  | (10531.4-13906.5) | (3321.6-4360.4) | (-0.50- -0.45) |  |
|  | Female | 3971.3 | 1338.7 | -0.48 |  | 11891.1 | 4010.9 | -0.47 |  |
|  |  | (3446.9-4541.3) | (1165.5-1522.4) | (-0.50- -0.45) |  | (10309.2-13585.5) | (3500.2-4574.8) | (-0.50- -0.45) |  |
|  | Eastern Sub-Saharan Africa | |  |  |  |  |  |  |  |
|  | Both | 10357.2 | 2356.7 | -0.31 |  | 31102.9 | 7035.4 | -0.31 |  |
|  |  | (8838.8-12178.3) | (2062.6 -2683.2) | (-0.34- -0.28) |  | (26568.4-36462.8) | (6138.0-8037.2) | (-0.34- -0.28) |  |
|  | Male | 4984.8 | 2283.5 | -0.27 |  | 14974.3 | 6814.5 | -0.28 |  |
|  |  | (4236.9-5874.1) | (1994.8-2593.6) | (-0.31- -0.24) |  | (12792.6-17570.3) | (5938.2-7776.2) | (-0.31- -0.25) |  |
|  | Female | 5372.4 | 2428.6 | -0.34 |  | 16128.6 | 7252.6 | -0.34 |  |
|  |  | (4575.1-6306.9) | (2126.2-2772.7) | (-0.37- -0.31) |  | (13792.9-18878.0) | (6337.2-8294.2) | (-0.37- -0.31) |  |
|  | Western Sub-Saharan Africa | |  |  |  |  |  |  |  |
|  | Both | 9252.1 | 1906.6 | -0.15 |  | 27773.9 | 5689.3 | -0.16 |  |
|  |  | (7885.5-10862.0) | (1665.3-2169.9) | (-0.19- -0.11) |  | (23734.7-32452.8) | (4960.2-6502.9) | (-0.20- -0.12) |  |
|  | Male | 4367.6 | 1834.8 | -0.15 |  | 13137.1 | 5480.9 | -0.15 |  |
|  |  | (3713.4-5113.2) | (1605.3-2085.5) | (-0.19- -0.10) |  | (11180.4-15389.3) | (4779.3-6258.2) | (-0.19- -0.11) |  |
|  | Female | 4884.5 | 1975.3 | -0.15 |  | 14636.8 | 5888.7 | -0.16 |  |
|  |  | (4177.5-5716.1) | (1721.8-2249.2) | (-0.20- -0.11) |  | (12515.4-17148.7) | (5158.9-6714.5) | (-0.20- -0.12) |  |
|  | Southern Sub-Saharan Africa | |  |  |  |  |  |  |  |
|  | Both | 1283.2 | 1605.8 | -0.30 |  | 3866.7 | 4842.5 | -0.30 |  |
|  |  | (1109.9-1470.5) | (1396.5-1828.7) | (-0.31- -0.28) |  | (3351.1-4456.4) | (4225.9-5523.4) | (-0.32- -0.28) |  |
|  | Male | 619.8 | 1573.8 | -0.30 |  | 1868.2 | 4746.7 | -0.30 |  |
|  |  | (532.9-713.7) | (1366.1-1799.0) | (-0.32- -0.27) |  | (1616.0-2153.9) | (4143.1-5425.2) | (-0.32- -0.28) |  |
|  | Female | 663.4 | 1636.2 | -0.30 |  | 1998.5 | 4933.6 | -0.30 |  |
|  |  | (577.6-757.7) | (1429.3-1861.2) | (-0.31- -0.28) |  | (1735.5-2295.3) | (4301.5-5633.2) | (-0.31- -0.28) |  |
|  | Central Sub-Saharan Africa | |  |  |  |  |  |  |  |
|  | Both | 2335.5 | 1733.4 | 0.03 |  | 7062.9 | 5210.8 | 0.01 |  |
|  |  | (1977.4-2736.7) | (1511.8-1972.3) | (0.01-0.07) |  | (6026.3-8265.7) | (4524.3-5947.8) | (-0.01-0.03) |  |
|  | Male | 1167.2 | 1741.3 | 0.02 |  | 3530.2 | 5233.7 | 0.00 |  |
|  |  | (991.5-1369.3) | (1517.6-1988.4) | (0.00-0.05) |  | (3009.1-4134.1) | (4556.1-5988.0) | (-0.02-0.03) |  |
|  | Female | 1168.3 | 1725.9 | 0.04 |  | 3532.7 | 5189.0 | 0.02 |  |
|  |  | (987.3-1365.5) | (1494.9-1968.7) | (0.01-0.07) |  | (3003.3-4139.8) | (4496.9-5955.7) | (-0.01-0.05) |  |
|  | High-income North America | |  |  |  |  |  |  |  |
|  | Both | 1725.4 | 536.6 | 0.46 |  | 5153.8 | 1608.7 | 0.48 |  |
|  |  | (1588.9-1873.9) | (489.4-590.3) | (0.34-0.58) |  | (4701.2-5613.2) | (1449.8-1771.7) | (0.36-0.59) |  |
|  | Male | 818.0 | 519.3 | 0.53 |  | 2443.1 | 1556.2 | 0.54 |  |
|  |  | (750.7-892.6) | (473.1-573.5) | (0.40-0.65) |  | (2225.3-2677.4) | (1400.3-1719.6) | (0.42-0.66) |  |
|  | Female | 907.4 | 553.2 | 0.39 |  | 2710.7 | 1658.7 | 0.41 |  |
|  |  | (837.7-983.1) | (504.7-605.3) | (0.27-0.50) |  | (2469.5-2942.6) | (1499.7-1823.3) | (0.30-0.53) |  |
|  | Tropical Latin America | |  |  |  |  |  |  |  |
|  | Both | 8027.9 | 3812.7 | -0.17 |  | 24141.2 | 11511.7 | -0.17 |  |
|  |  | (7056.2-9091.5) | (3338.7-4354.9) | (-0.30- -0.03) |  | (21169.4-27463.7) | (10067.7-13175.0) | (-0.30- -0.04) |  |
|  | Male | 3804.8 | 3655.0 | -0.16 |  | 11447.4 | 11038.3 | -0.16 |  |
|  |  | (3339.4-4314.1) | (3197.6-4167.9) | (-0.29- -0.03) |  | (10046.0-13030.1) | (9662.9-12644.1) | (-0.29- -0.03) |  |
|  | Female | 4223.1 | 3967.5 | -0.17 |  | 12693.9 | 11976.6 | -0.18 |  |
|  |  | (3715.7-4786.0) | (3472.6-4543.6) | (-0.31- -0.04) |  | (11157.1-14457.4) | (10492.8-13699.9) | (-0.31- -0.05) |  |
|  | Andean Latin America | |  |  |  |  |  |  |  |
|  | Both | 1409.9 | 2255.1 | -0.30 |  | 4260.2 | 6813.8 | -0.30 |  |
|  |  | (1225.2-1610.4) | (1964.0-2574.6) | (-0.31- -0.29) |  | (3705.3-4888.4) | (5955.1-7785.5) | (-0.31- -0.29) |  |
|  | Male | 698.8 | 2219.9 | -0.30 |  | 2111.8 | 6706.3 | -0.30 |  |
|  |  | (606.9-801.5) | (1938.9-2535.9) | (-0.31- -0.29) |  | (1835.3-2425.9) | (5858.6-7688.8) | (-0.32- -0.29) |  |
|  | Female | 711.1 | 2290.5 | -0.30 |  | 2148.4 | 6921.6 | -0.30 |  |
|  |  | (618.2-813.2) | (1995.6-2610.5) | (-0.30- -0.29) |  | (1870.0-2461.7) | (6039.1-7911.8) | (-0.30- -0.29) |  |
|  | Central Latin America |  |  |  |  |  |  |  |  |
|  | Both | 4015.5 | 1558.0 | -0.30 |  | 12109.9 | 4705.4 | -0.30 |  |
|  |  | (3478.4-4577.5) | (1352.5-1771.1) | (-0.32- -0.27) |  | (10505.9-13829.1) | (4098.9-5373.5) | (-0.33- -0.27) |  |
|  | Male | 2015.9 | 1579.4 | -0.30 |  | 6081.4 | 4769.6 | -0.30 |  |
|  |  | (1745.3-2303.7) | (1371.9-1795.9) | (-0.33- -0.26) |  | (5278.6-6958.6) | (4163.1-5442.0) | (-0.33- -0.26) |  |
|  | Female | 1999.6 | 1536.8 | -0.30 |  | 6028.5 | 4642.0 | -0.30 |  |
|  |  | (1734.7-2280.3) | (1336.0-1748.7) | (-0.32- -0.27) |  | (5231.1-6904.0) | (4050.4-5307.0) | (-0.32- -0.27) |  |
|  | Southern Latin America | |  |  |  |  |  |  |  |
|  | Both | 175.6 | 279.9 | -0.53 |  | 527.3 | 843.3 | -0.51 |  |
|  |  | (152.6-200.9) | (241.6-321.8) | (-0.60- -0.46) |  | (459.5-602.3) | (731.2-967.7) | (-0.59- -0.44) |  |
|  | Male | 85.4 | 277.2 | -0.62 |  | 256.4 | 834.6 | -0.60 |  |
|  |  | (73.5-98.4) | (238.1-321.5) | (-0.69- -0.55) |  | (221.6-294.7) | (719.1-962.3) | (-0.67- -0.54) |  |
|  | Female | 90.2 | 282.2 | -0.45 |  | 270.9 | 850.4 | -0.43 |  |
|  |  | (78.8-102.7) | (244.6-324.3) | (-0.53- -0.36) |  | (237.2-309.2) | (741.6-975.5) | (-0.51- -0.35) |  |
|  |  |  |  |  |  |  |  | |  |

ASR, age standardized rate (per 100,000 population); CI, conﬁdence interval; UI, uncertainty interval; SDI, socio-demographic index.

Data from a total of 195 countries and territories were available. Geographically, the world was classified as 21 regions, on the basis of geographical proximity, sociocultural, and epidemiological similarities. The Socio-demographic Index (SDI) is a summary measure of development status strongly correlated with health outcomes. According to the SDI 195 countries and territories were separated into 5 regions with low, low-middle, middle, high-middle, and high SDI.


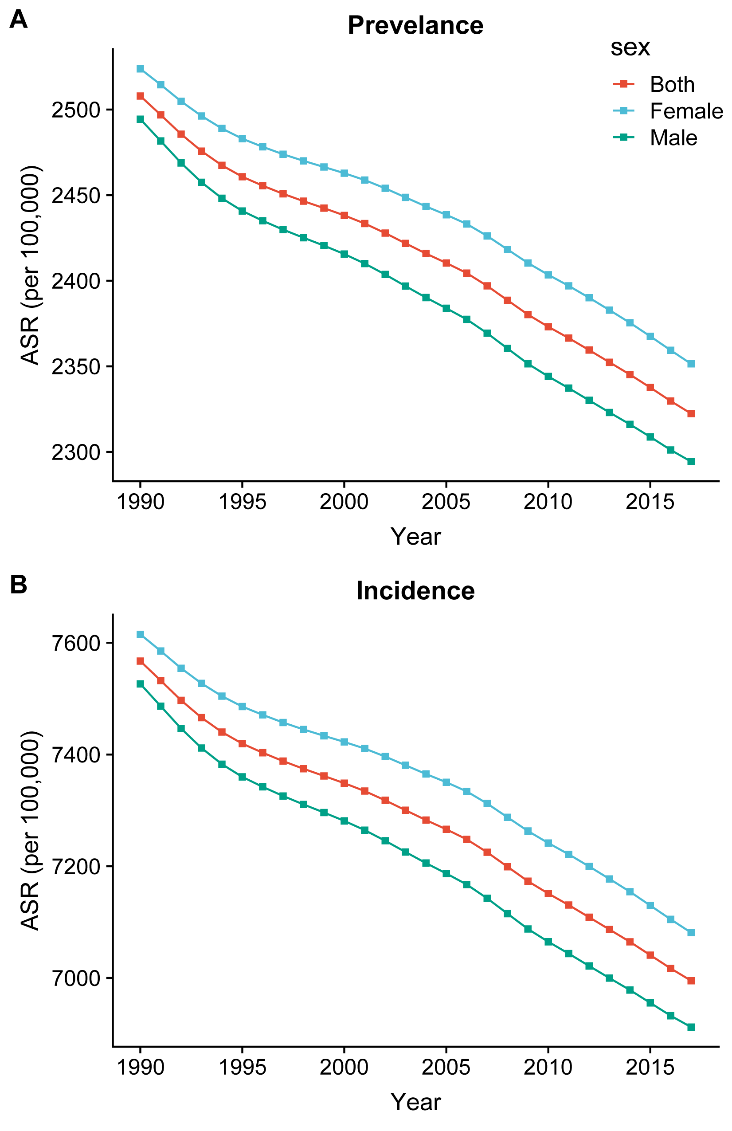


**Figure S1. The global age standardized rate (ASR) of scabies prevalence (A) and incidence (B), 1990-2017.**
